# Differential localization of glioblastoma subtype: implications on glioblastoma pathogenesis

**DOI:** 10.18632/oncotarget.8551

**Published:** 2016-04-01

**Authors:** Tyler C. Steed, Jeffrey M. Treiber, Kunal Patel, Valya Ramakrishnan, Alexander Merk, Amanda R. Smith, Bob S. Carter, Anders M. Dale, Lionel M. L. Chow, Clark C. Chen

**Affiliations:** ^1^ Center for Theoretical and Applied Neuro-Oncology, Division of Neurosurgery, Moores Cancer Center, University of California, San Diego, La Jolla, CA, USA; ^2^ Weill-Cornell Medical College, New York Presbyterian Hospital, New York, NY, USA; ^3^ Cancer and Blood Diseases Institute, Cincinnati Children's Hospital Medical Center, Cincinnati, OH, USA; ^4^ Multimodal Imaging Laboratory, University of California San Diego, La Jolla, CA, USA; ^5^ Department of Radiology, University of California San Diego, La Jolla, CA, USA

**Keywords:** glioblastoma, MR imaging, subventricular zone, subtypes, automatic tumor segmentation

## Abstract

**Introduction:**

The subventricular zone (SVZ) has been implicated in the pathogenesis of glioblastoma. Whether molecular subtypes of glioblastoma arise from unique niches of the brain relative to the SVZ remains largely unknown. Here, we tested whether these subtypes of glioblastoma occupy distinct regions of the cerebrum and examined glioblastoma localization in relation to the SVZ.

**Methods:**

Pre-operative MR images from 217 glioblastoma patients from The Cancer Imaging Archive were segmented automatically into contrast enhancing (CE) tumor volumes using Iterative Probabilistic Voxel Labeling (IPVL). Probabilistic maps of tumor location were generated for each subtype and distances were calculated from the centroid of CE tumor volumes to the SVZ. Glioblastomas that arose in a Genetically Modified Murine Model (GEMM) model were also analyzed with regard to SVZ distance and molecular subtype.

**Results:**

Classical and mesenchymal glioblastomas were more diffusely distributed and located farther from the SVZ. In contrast, proneural and neural glioblastomas were more likely to be located in closer proximity to the SVZ. Moreover, in a *GFAP-CreER*; *Pten^loxP/loxP^*; *Trp53^loxP/loxP^*; *Rb1^loxP/loxP^*; *Rbl1^−/−^* GEMM model of glioblastoma where tumor can spontaneously arise in different regions of the cerebrum, tumors that arose near the SVZ were more likely to be of proneural subtype (*p* < 0.0001).

**Conclusions:**

Glioblastoma subtypes occupy different regions of the brain and vary in proximity to the SVZ. These findings harbor implications pertaining to the pathogenesis of glioblastoma subtypes.

## INTRODUCTION

Glioblastoma remains one of the deadliest of human cancers [1]. Large-scale genomic analyses of clinical glioblastoma samples have identified at least five subtypes, each with distinct biologic and clinical behaviors [2-5]. These subtypes include classical, mesenchymal, neural, proneural, and the glioma-CpG island methylator phenotype (G-CIMP). Intriguingly, in Genetically Modified Murine Model (GEMM) of glioblastoma, the same set of cancer causing mutations can give rise to multiple glioblastoma subtype [6]. It remains unclear whether these molecular differences arise as a result of distinct cells of origin, epigenetic landscape, or microenvironment.

The sub-ventricular zone (SVZ), which lies adjacent to the lateral wall of the lateral ventricle, is a site where neural stem cells (NSC) and astrocyte precursors are located in the adult brain [7-9]. During neural development, NSCs migrate radially and differentiate into various progenitor cells during this process [10-12]. NSCs and progenitor cells are, thus, located at varying distance from the SVZ. The available data suggest that mutations occurring in these cell populations may give rise to glioblastomas [13, 14]. If the cell of origin contributes toward subtype-specific pathogenesis, then one would expect clustering of select subtypes with respect to distance from the SVZ. Here we tested this hypothesis.

Using Iterative Probabilistic Voxel Labeling (IPVL) [15], a method of automatic tumor segmentation developed by our laboratory, and imaging data obtained through The Cancer Imaging Archive (TCIA), we quantitatively determined the geographic distribution for each of the molecular glioblastoma subtypes and quantified each tumor's distance to the SVZ. Our analysis indicates that the proneural and neural glioblastoma subtypes are located in close proximity to SVZ. In a *GFAP-CreER*; *Pten^loxP/loxP^*; *Trp53^loxP/loxP^*; *Rb1^loxP/loxP^*; *Rbl1^−/−^* Genetically Modified Murine Model (GEMM) of glioblastoma where tumor can spontaneously arise in different regions of the cerebrum [16], glioblastoma that arose near the SVZ were more likely to be of proneural subtype. These findings provide clinical evidence that subtype biology may be related to differing cells of origin.

## RESULTS

### Glioblastoma density map

**Figure 1:**
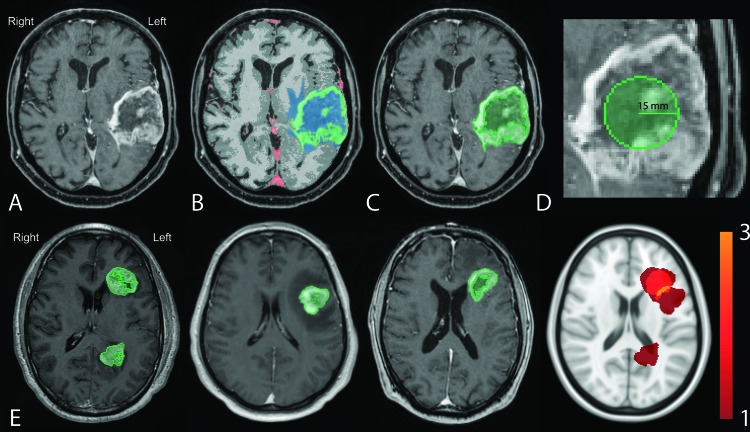
Workflow for generation of total CE and centroid density maps Preprocessed images **A.** were registered to the Montreal Neurological Institute (MNI) template and segmented according to the IPVL pipeline **B.** CE volumes were filled **C.** and centroid of each were calculated **D. E.** Filled CE volumes from each subject (first three panels) were summed (right most panel) and then converted to tumor density map.

**Figure 2:**
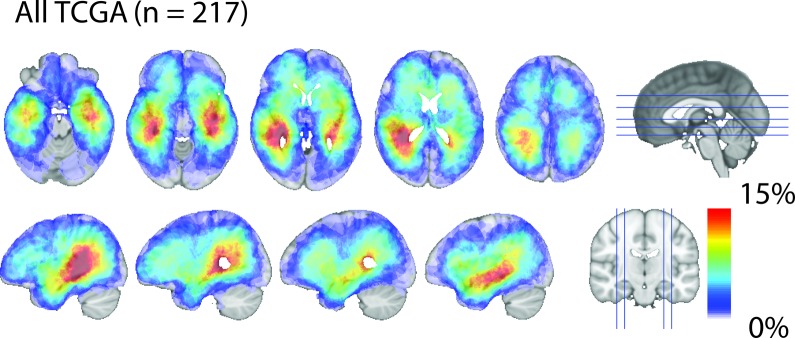
Glioblastoma density map Total CE probability map revealed that glioblastoma, as a whole, exhibit a strong predilection occurrence in proximity to the SVZ. Red indicates the highest frequency of overlap and light-blue indicating the lowest frequency of overlap.

The workflow for CE segmentation and CE centroid placement is described in Methods and shown in Figure 1. Information regarding patient demographics, patient survival, and tumor CE volumes for each patient are available in Table 1. The filled CE volume from all subjects are layered and generated in template space yielding a glioblastoma density map and shown as Figure 2. The centroid of CE volume for each patient was similarly layered to create a density map, which confirmed the same distribution (Supplemental Figure 1). Glioblastoma tumor densities are enriched around the SVZ. 25.7% of all CE volume probabilities were found within 10 mm of the ventricular volume, a region representing only 12.6% percent of the total brain volume. This finding is largely consistent with two previous publications [17, 18].

**Table 1:** Demographic data

Variable	All	G-CIMP+ Proneural	G-CIMP- Proneural	Neural	Classical	Mesenchymal	Unknown
N (%)	217 (100)	10 (4.6)	46 (21.2)	39 (18.0)	51 (23.5)	57 (26.3)	14 (6.5)
Male, No. (%)	128 (59.8)	6 (66.7)	25 (55.6)	25 (64.1)	23 (45.1)	39 (68.4)	10 (76.9)
Female, No. (%)	86 (40.2)	3 (33.3)	20 (44.4)	14 (35.9)	28 (54.9)	18 (31.6)	3 (23.1)
Age, y, mean ± SD	59.3 ± 14.1	41.5 ± 15.4	57.9 ± 15.1	62.0 ± 13.3	61.2 ± 15.0	60.7 ± 11.2	56.6 ± 12.0
KPS, mean ± SD	77.9 ± 14.1	86.7 ± 10.0	76.6 ± 9.9	78.2 ± 19.3	75.0 ± 14.3	79.6 ± 13.4	75.5 ± 16.3
OS, d, mean ± SD	405.8 ± 336.0	680.8 ± 287.1	320.4 ± 343.2	427.8 ± 300.9	433.6 ± 291.8	429.0 ± 388.2	197.0 ± 159.5
PFS, d, mean ± SD	261.2 ± 271.8	441.6 ± 593.0	241.9 ± 315.9	255.2 ± 189.2	241.0 ± 169.2	294.1 ± 308.3	151.5 ± 118.2
CE volume, mean ± SD	34977 ± 33406	26809 ± 12454	29015 ± 18364	40504 ± 37324	32496 ± 20217	39371 ± 49242	35146 ± 25987

CE: contrast enhancing; SD: standard deviation

### Glioblastoma subtype specific density maps

**Figure 3:**
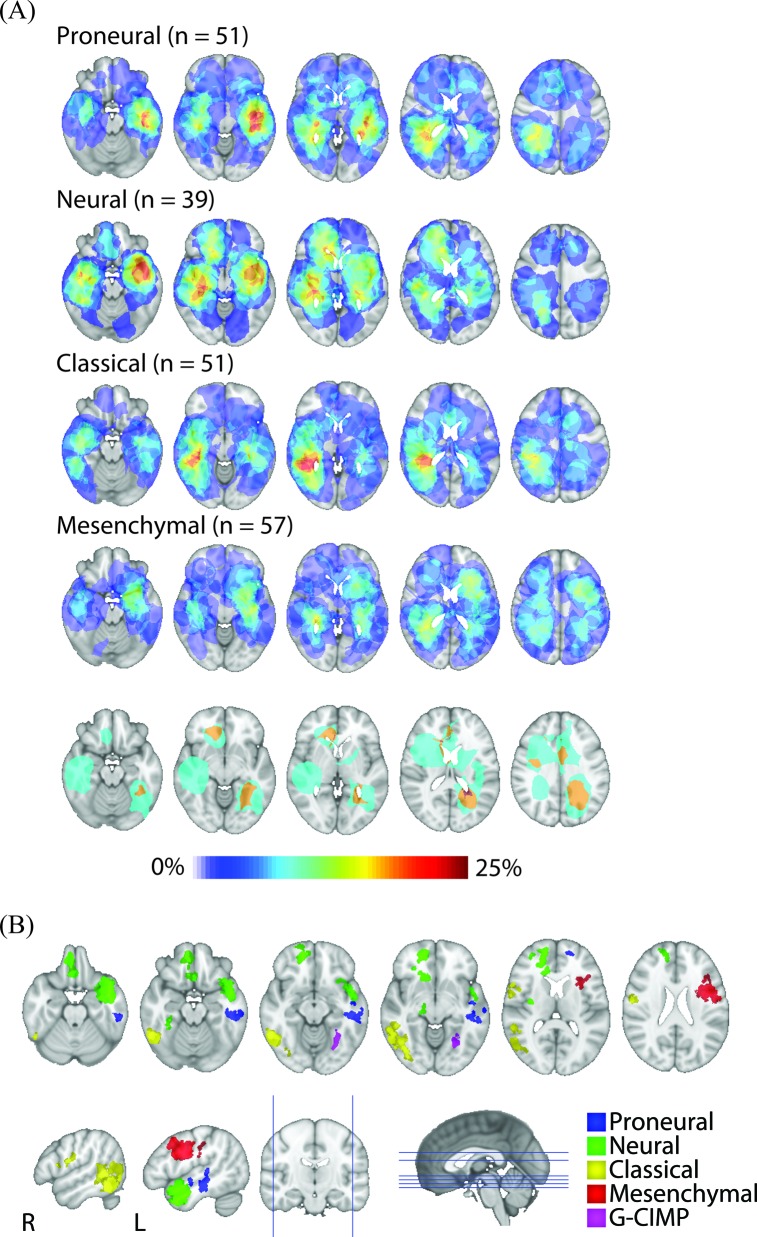
Glioblastoma subtype density maps and regions of statistically significant subtype localization **A.** Subtype-specific density maps were generated using total CE volume. Red indicates the highest frequency of overlap and light-blue indicating the lowest frequency of overlap. Proneural and neural tumors tend to occur in the temporal and frontal lobe, having higher densities in the left temporal region relative to the right. In contrast, the classical and mesenchymal subtypes were more diffusely distributed in the cerebrum, with significantly lower probabilities of overlap (*p* < 0.001). **B.** Axial (first row) and sagittal (second row) of statistically significant clusters (*p* < 0.05) by subtype. Statistical comparisons were carried out using voxel-wise Fisher's exact tests.

We next generated CE density maps for each glioblastoma subtype. We observed that proneural and neural tumors tend to occur in the temporal and frontal lobe. Both of these subtypes demonstrated a predilection towards asymmetry, having higher densities in the left temporal region relative to the right (Figure 3A). In contrast, the classical and mesenchymal subtypes were more diffusely distributed in the cerebrum, with significantly lower probabilities of overlap (*p* < 0.001) (Figure 3A). The classical subtype had higher probability densities within the periventricular white matter adjacent to the right atrium. Statistical comparisons between the probability density maps for all subtypes using voxel-wise Fisher's exact testing (see Methods) confirmed the distributions described above demonstrating regions that are highly specific for each subtype. The statistically significant regions associated with each subtype are demonstrated by Figure 3B. The limited number of G-CIMP glioblastomas (*n* = 11) did not exhibit specific geographic association to any particular regions of the cerebrum.

### Glioblastoma subtypes and SVZ distance

**Figure 4:**
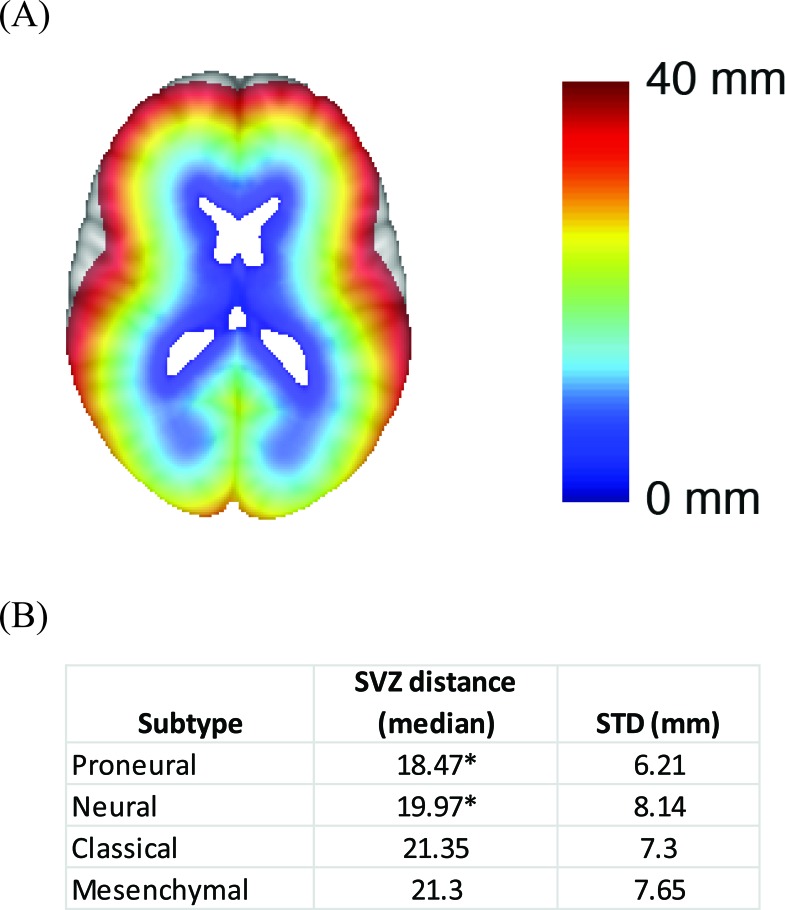
Association between SVZ distance and glioblastoma subtype **A.** Graphical illustration of the SVZ distance measurement. SVZ distance is color coded so that blue indicates shorter SVZ distances while red indicates high SVZ distances. **B.** Median SVZ distances for the glioblastoma subtypes. * indicates statistical significance based on student's t-test when comparing the SVZ distance of the proneural subtype to those of the classical or the mesenchymal subtype. The same comparisons were performed for the neural subtype.

To study the anatomic relation of glioblastoma subtype to the SVZ, we developed a quantitative measure of SVZ distance (See Methods). An illustrative example of the SVZ distance measure is demonstrated in Figure 4A. Applying this measure to our dataset, we found that proneural and neural subtypes were located in closer proximity to the SVZ relative to the mesenchymal and classical subtypes (*p* = 0.035) (Figure 4B). Since clinical studies of glioblastoma growth based on serial MRI images suggest that the center of tumor mass approximates the site of tumor origin [19], our results suggest that glioblastoma subtypes arise in distinct regions of the cerebrum.

**Figure 5:**
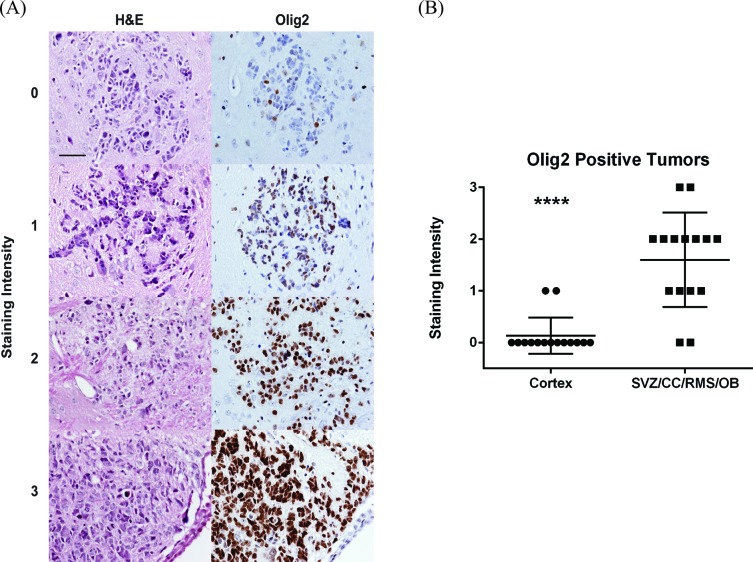
Analysis of glioblastoma location in a GEMM by subtype The location and Olig2 staining characteristics of early glioblastomas were examined in GEMM model of glioblastoma [16]. **A.** Tumors were identified based on H&E staining (left column) and an adjacent slide stained for Olig2 (right column). Representative images demonstrating Olig2 staining intensity from 0 to 3 are shown. Scale bar in the upper right hand image is 50 μm and applies to all images. **B.** Olig2 staining of cortical tumors was compared to tumors of the subventricular zone (SVZ), overlying corpus callosum (CC), rostral migratory stream (RMS) and olfactory bulb (OB) in this scatter plot. Lines represent the mean and standard deviation. ****p < 0.0001.

To confirm our clinical finding, we tested the distribution of glioblastoma subtypes in a *GFAP-CreER*; *Pten^loxP/loxP^*; *Trp53^loxP/loxP^*; *Rb1^loxP/loxP^*; *Rbl1^−/−^* GEMM model where glioblastoma can arise anywhere in the cerebrum [16]. The brains of the mice were sectioned in their entirety and examined for the presence of early glioblastomas, in particular recording their location. For this study, we focused on tumors located in the cortex (external to the corpus callosum and external capsule) and those in close proximity to or extending from the SVZ (including the overlying corpus callosum, rostral migratory stream and olfactory bulb). We characterized Olig2 staining intensity, an established biomarker for proneural glioblastoma [2, 20-22], for each tumor (Figure 5A). The glioblastomas that arose from the GEMM showed a wide range of Olig2 staining intensities, which we graded from a scale of 0 to 3+. Nearly all tumors staining of 2+ and 3+ were classified as proneural or neural subtype when mRNA profiling of these tumors were performed [6]. In contrast, tumors staining 0-1 were classified as mesenchymal or classical subtype. Consistent with our analysis of TCIA, the proneurral/neural glioblastomas (2+ and 3+ Olig 2 staining glioblastomas) tend to locate in proximity to the SVZ. In contrast, the mesenchymal or classical glioblastomas (0 and 1+ Olig 2 staining glioblastomas) were more likely located in the cortex, distant from the SVZ (Figure 5B; *p* < 0.0001). These observations in the GEMM model confirm our clinical observation from human subjects that proneural glioblastomas are more likely to occur in proximity to the SVZ.

## DISCUSSION

Our study provides a quantitative tumor density map of glioblastoma subtypes and demonstrates that these subtypes occupy distinct regions of the brain. Intriguingly, the subtypes exhibit regional associations with regard to the SVZ, an area of critical importance in neurogenesis and glioblastoma pathogenesis. The proneural and neural glioblastomas exhibit anatomic tropism reminiscent of NSCs (in proximity to the SVZ) and show gene expression patterns resembling these cells [2, 22]. In contrast, the classical and mesenchymal glioblastomas exhibit a more diffuse distribution and are found farther from the SVZ - a distribution that is similar to those of neural progenitor cells. We further confirmed that in a GEMM model where glioblastoma can spontaneous arise in distinct regions of the cerebrum, proneural glioblastomas are more likely to arise in proximity to the SVZ. These findings provide clinical evidence that subtype biology may be related to differing cells of origin.

The asymmetric distribution of proneural and neural subtypes presents an unexpected phenomenon. A large body of work has documented asymmetry in cerebral function and activity [23], with inferred asymmetry in terms of anatomic substrate and physiologic circuit [24-26]. It is possible that molecular microenvironment or differential cell-related asymmetry contributes to glioblastoma pathogenesis. For instance, recent studies suggest that many neurotransmitters serve as trophic factors that mediate mitogenic signaling in glioblastomas [27]. Increased utilization of such neurotransmitters [28], or differential levels of adult neurogenesis may effect regional predisposition for glioblastoma pathogenesis. As an alternative explanation, patients with tumors affecting the left temporal lobe may present with symptoms earlier, potentially increasing their likelihood for detection and enrollment in the TCGA.

Amongst the limitations of our study is the assumption that the center of tumor mass approximates the site of tumor origin. This assumption is largely supported by mathematical studies that use imaging to model the growth pattern of glioblastomas [19]. Another challenge encountered during our study involves the deformation of ventricular contour by glioblastoma mass effect. To address this issue, we utilized established methods of registration [27] to MNI atlas to account for such deformations. Finally, while robust statistical analysis is performed in this dataset in a hypothesis-driven manner, further verification of our results is warranted.

In conclusion, the integration of quantitative MR image analysis of 217 TCIA subjects and molecular subtyping of glioblastoma revealed subtype specific brain localization and differed with regard to the SVZ. Quantitative proximity to the SVZ, as determined by our methods, may be an imaging proxy for the underlying glioblastoma biology.

## MATERIALS AND METHODS

### Data acquisition and subtype classification

We searched the TCIA for subjects with at least one artifact-free pre-operative T1 weighted MR image with contrast. In total, 217 subjects with pre-surgical resection MR images were downloaded from the TCIA (http://cancerimagingarchive.net) in November 2014. Level 3 probe collapsed Messenger RNA (mRNA) expression data: Affymetrix HT-HG-U133A GeneChip and RNAseq, was downloaded for available patients *via* the TCGA Data Portal (http://tcga-data.nci.nih.gov/tcga/). Affymetrix expression data were normalized by robust multichip average (RMA). RNAseq data were RSEM normalized. When not already available in published literature, genomic subtypes were determined for subjects employing single sample Gene Set Enrichment Analysis (ssGSEA) as described previously [29, 30]. Additionally, G-CIMP status was determined using methods described previously [3, 30].

### Image preprocessing and registration

MR images were corrected for gradient nonlinearity using previously described methods [18, 31]. Images were additionally preprocessed and intensity corrected utilizing N4 bias field correction [32], then registrations were performed to the Montreal Neurological Institute (MNI) 152 1 mm^3^ template employing methods from Advanced Normalization Tools (ANTS) [27]. Visual inspections were subsequently performed by three independent reviewers (T.C.S, J.M.T, & K.S.P) to ensure accurate preprocessing. Variability in the technical parameters of the images analyzed is described in our previous study [15].

### Tumor segmentation and probability maps

Contrasting-enhancing (CE) regions of tumors were segmented using the iterative probabilistic voxel labeling (IPVL) algorithm developed in the laboratory [15] (Figure 1B) and the enclosed volume was filled (See Methods, Figure 1C). Tumor density maps were calculated as the number of observations at each voxel divided by the number of subjects (Figure 1E). Similarly, centroid density maps were generated using the regionprops(matrix,’centroid’); function in MATLAB (The MathWorks, Inc., Natick, Massachusetts, United States.) where a 15-mm sphere placed at the center of mass for each filled CE volume (Figure 1D). Visual inspection by two independent operators (T.C.S, J.M.T) was performed to ensure adequacy of region filling and centroid estimation. Probability distributions were quantitatively compared using a voxel-wise two-tailed Fisher's exact test [33] comparing the number of tumors observed for each subtype at each voxel. Only significant clusters which had a >5% probability of occurring by chance were kept.

### Subventricular zone distance

To measure SVZ distance with respect to each tumor's filled CE volume, the MNI template's lateral ventricle segmentation was used as a basis of comparison. Linear transformation of the glioblastoma volume to the MNI template was performed. SVZ distances were calculated by taking the mean of the distance from the nearest MNI template ventricular border to each point within a subject's CE volume.

### GEMM model of glioblastoma

Quadruple conditional knock-out (cKO) mice have been described elsewhere [16]. Briefly, the genotype of the mice used in this study is *GFAP-CreER*; *Pten^loxP/loxP^*; *Trp53^loxP/loxP^*; *Rb1^loxP/loxP^*; *Rbl1^−/−^*. Deletion of conditional alleles was induced in five week old mice by intraperitoneal tamoxifen injections, 9 mg/40 g body weight daily for three days. Six weeks after tamoxifen injections, mice were euthanized by transcardial perfusion with phosphate buffered saline (PBS). The brain was dissected, cut sagittally down the midline and fixed by immersion in 4% paraformaldehyde in PBS for 12 hrs. The tissue was processed for paraffin embedding and 5 μm thin sections prepared. Every fifth slide was stained with hematoxylin and eosin to identify and localize tumors, which were confirmed by immunohistochemical staining for the proliferation marker, Ki67. To characterize the tumor, selected adjacent slides were stained for Olig2 (dilution 1:500; EMD Millipore, Temecula, CA). Staining intensity was graded as absent or weak (0), positive in >50% of tumor cells (1), positive in >90% of tumor cells (2) or strongly positive (3). Average staining intensity in tumors from each location was calculated and significance determined using the two-tailed t-test.

## SUPPLEMENTARY MATERIAL FIGURE



## Abbreviations

T1wCE: T1WI with Contrast
CEV: Contrast-Enhancing Volume
FHV: FLAIR Hyperintensity Volume
SVZ: Subventricular Zone
G-CIMP: CpG Island Methylator Phenotype
TCGA: The Cancer Genome Atlas
TCIA: The Cancer Imaging Archive
REMBRANDT: Repository for Molecular Brain Neoplasia Data
