# Supplementary material for: Differential localization of glioblastoma subtype: implications on glioblastoma pathogenesis
Source: Oncotarget. 2016 Apr 1;7(18):24899–907. doi: 10.18632/oncotarget.8551 (PMC5041878; doi:10.18632/oncotarget.8551)
Supplement: Supplementary file 1 [file oncotarget-07-24899-s001.pdf]

Differential localization of glioblastoma subtype: implications on glioblastoma pathogenesis

Supplementary Material

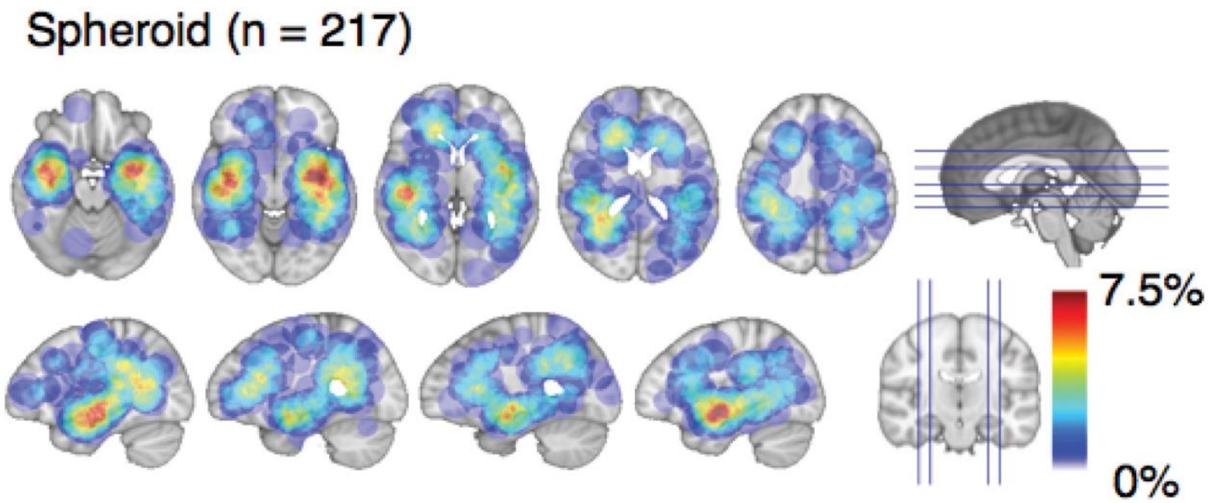

Supplemental Figure 1. Glioblastoma subtype density maps. Subtype-specific density maps were generated using a 15-mm centroid of each filled CE volume. Red indicates the highest frequency of overlap and light-blue indicating the lowest frequency of overlap. Proneural and neural tumors tend to occur in the temporal and frontal lobe, having higher densities in the left temporal region relative to the right. In contrast, the classical and mesenchymal subtypes were more diffusely distributed in the cerebrum, with significantly lower probabilities of overlap ($p < 0.001$)
